# Therapeutic effect of sodium alginate on bleomycin, etoposide and cisplatin (BEP)-induced reproductive toxicity by inhibiting nitro-oxidative stress, inflammation and apoptosis

**DOI:** 10.1038/s41598-024-52010-w

**Published:** 2024-01-18

**Authors:** Mojtaba Moradi, Mohammad Arshia Hashemian, Azita Faramarzi, Nader Goodarzi, Amir Hossein Hashemian, Hadi Cheraghi, Cyrus Jalili

**Affiliations:** 1 Department of Clinical Sciences, Faculty of Veterinary Medicine, Razi University, Kermanshah, Iran; 2 Fertility and Infertility Research Center, Health Technology Institute, Kermanshah University of Medical Sciences, Kermanshah, Iran; 3 Department of Anatomical Sciences, Medical School, Kermanshah University of Medical Sciences, Kermanshah, Iran; 4 Department of Basic and Pathobiological Sciences, Faculty of Veterinary Medicine, Razi Universtiy, Kermanshah, Iran; 5 Research Center for Environmental Determinants of Health (RCEDH), Health Institute, Kermanshah University of Medical Sciences, Kermanshah, Iran; 6 Department of Biostatistics, School of Health, Kermanshah University of Medical Sciences, Kermanshah, Iran; 7 Medical Biology Research Center, Health Technology Institute, Kermanshah University of Medical Sciences, Kermanshah, Iran

**Keywords:** Cancer, Chemical biology, Drug discovery

## Abstract

Impaired spermatogenesis and male infertility are common consequences of chemotherapy drugs used in patients with testicular cancer. The present study investigated the effects of sodium alginate (NaAL) on testicular toxicity caused by bleomycin, etoposide, and cisplatin (BEP). Rats in group 1 received normal saline, while groups 2 and 3 were treated with 25 and 50 mg/kg of NaAL, respectively. Group 4 was treated with a 21-day cycle of BEP (0.5 mg/kg bleomycin, 5 mg/kg etoposide, and 1 mg/kg cisplatin), and groups 5 and 6 received BEP regimen plus 25 and 50 mg/kg of NaAL, respectively. Then, sperm parameters, testosterone levels, testicular histopathology and stereological parameters, testicular levels of malondialdehyde (MDA), nitric oxide (NO), and total antioxidant capacity (TAC), and the expression of apoptosis-associated genes including Bcl2, Bax, Caspase3, p53, and TNF-α were evaluated. Our findings revealed that NaAL improved sperm parameters, testosterone levels, histopathology, and stereology parameters in BEP-administrated rats. NaAL also improved testis antioxidant status by enhancing TAC and ameliorating MDA and NO. Further, modifications to the expression of Bcl2, Bax, Caspase3, p53, and TNF-α suggested that NaAL alleviated BEP-induced apoptosis and inflammation. Collectively, NaAL protects rats’ testes against BEP-evoked toxicity damage through the modulation of nitro-oxidative stress, apoptosis, and inflammation.

## Introduction

Cancer and its treatment, including anticancer drugs and chemotherapy regimens, are one of the most serious health issues^1^. Even though chemotherapy and anticancer drugs have demonstrated various successes in improving cancer patients' quality of life, they have exhibited a wide range of adverse effects^1,2^.

Cisplatin-based chemotherapy regimens, especially in combination with bleomycin and etoposide, are used against many types of cancer, especially testicular tumors^3–5^. The most common malignancy affecting young adult males aged between 20 and 35 years is testis tumors, including germ cell and stromal cell tumors^6–8^. Co-administration of bleomycin, etoposide, and cisplatin (BEP) regimen in three to four cycles of chemotherapy is globally accepted chemotherapy in patients with metastatic germ cell tumors, even in those with intermediate and poor prognosis^5,9–11^. With timely treatment, BEP is a highly reliable chemotherapy and can effectively cure around 85–90% of patients with metastatic disease and over 95% of those with favorable prognostic features without recurrence^4,6^. Moreover, the BEP regimen is highly recommended for treating high-risk, recurrent, or persistent gestational trophoblastic neoplasia, which is a spectrum of tumors that arise from placental tissue^12^. All three drugs in BEP interfere with DNA replication and chromatin structure and invade germ cells because of their high cell division capacity^5,13^.

The BEP regimen, however, carries a high risk of toxicity to various organs and tissues^14,15^. In this context, BEP-evoked testicular toxicity and dysfunction are the most problematic side effects of this cancer treatment and one of the most significant long-term complications for young cancer survivors^14,16–21^. BEP drugs are gonadotoxic and commonly induce a persistent decline in normal Leydig cell function, reduced androgen production, oligospermia, azoospermia, and decreased libido, resulting in an irreversible impairment of fertility^5,7,17,20–23^. This chemotherapy regimen also reduces sperm count and motility and induces apoptosis, seminiferous tubular atrophy, and adverse outcomes in offspring, which vary depending on the dosage and treatment period^5,7,18,24^.

Infertility is a social, cultural, and medical issue receiving widespread attention and affecting at least 8–12% of couples globally. The incidence of male factor infertility has increased significantly in recent years and is a primary cause of around 50% of couples' infertility^25–30^. Male infertility occurs for several reasons, including smoking, alcohol use, obesity, varicoceles, cancer, and cancer therapy^27,28,31^. The process of spermatogenesis and protecting spermatogonial stem cells is essential for human reproduction^32^. Since spermatogonial cells are the precursors of all the spermatogenic cells and the Sertoli cells, as supportive cells, they play pivotal roles in spermatogenesis, and the degree of damage to these cells determines the chance of fertility recovery^5^. Given that the treatment of infertility is not covered by essential health insurance and imposes a considerable economic burden on patients and the healthcare system, measures should be taken to protect fertility in cancer patients who receive chemotherapy drugs^27^.

While physiological levels of reactive oxygen species (ROS) are required for male fertility, particularly for acrosome reaction and capacitation, higher levels of ROS lead to oxidative stress and low sperm quality^31,33,34^. Oxidative stress as a common consequence of cancer treatments results in oxidative stress-related male sub/infertility^33^. Natural and synthetic antioxidants are widely used to address reproductive toxicity and male infertility caused by various toxicants and anticancer drugs^33,35^. In this regard, we recently demonstrated that melatonin protects testes' function and spermatogenesis from BEP chemotherapy regimen-induced reproductive toxicity via modulating nitro-oxidative stress, apoptosis, and inflammation^5^.

Sodium alginate (NaAL) is an anionic polysaccharide abundant in nature and occurs both as a structural component in marine brown algae and as a capsular polysaccharide in soil bacteria^36–38^. The sodium salt of alginic acid is extracted from brown algae and is an inexpensive medical material used in a variety of products, from food additives to pharmaceuticals^36^. Recently, it has been used as a bioengineering scaffold for tissue regeneration in regenerative medicine^36,39^. It has been reported that NaAL is used as a common drug adjuvant with antioxidant and immunoprotective properties. Moreover, alginate oligosaccharide is introduced as a non-allergenic, non-toxic, and biodegradable polymer with antioxidant, anti-inflammatory, anti-apoptotic, and anti-proliferative properties. Consequently, they are extensively used as carriers for drug delivery, immunoisolation systems for transplantation, wound dressing, and as a protective agent in radiotherapy^37,40–43^. In this vein, Kumar et al.^44^ revealed that supplementation of buffalo semen extender with NaAL led to improvements in antioxidant capacity, free radical scavenging, and metal reduction capacity during the preservation of sperm during cryopreservation. Moreover, Guo et al.^40^ indicated that alginate oligosaccharides mitigate myocardial reperfusion injury via modulating nitro-oxidative stress and endoplasmic reticulum stress-mediated apoptosis in mice. It has also been demonstrated that mouse testicular cells encapsulated with biomaterials such as NaAL self-organize into seminiferous tubules with blood-testis barrier formation and Leydig cell differentiation in vitro^45^. However, whether NaAL can protect against BEP chemotherapy regimen-induced testicular toxicity and minimize its toxicity has remained obscure.

Hence, this study aimed to investigate the alleviative effects of NaAL following BEP chemotherapy exposure on sperm quality, nitro-oxidative markers, histopathological structure, and stereological parameters, as well as inflammatory and apoptotic alternations in rat testes.

## Materials and methods

### Animals

Sixty adult male albino Wistar rats (250–300 g, 13–15 weeks old) were purchased from the Pasteur Institute (Tehran, Iran). The animals were habituated for a week and then divided into control and experimental groups. The animals were held under standard conditions, including a 12-h dark/12-h light period, 23 ± 1 °C temperature, and 50–60% humidity, and had free access to water and food (standard diet) throughout the study. All experiments were carried out in strict accordance with the international guidelines for the care and use of laboratory animals and were approved by the Animal Welfare and Ethics Committee of Razi University and the Kermanshah University of Medical Science, Iran (Approval no: R.KUMS.REC.1398.1033). All methods were carried out in accordance with ARRIVE guidelines and regulations (https://arriveguidelines.org).

### Experimental design and treatment protocols

The applied doses in human practice were converted to rat doses reflecting a 1× dose. Due to the high hepatotoxicity and especially nephrotoxicity of 1× dose for rats (according to our previous studies), the × 0.33 doses of the BEP regimen (0.5 mg/kg bleomycin, 5 mg/kg etoposide, and 1 mg/kg cisplatin) were used for the examined animals (according to our previous studies)^5,46^. The animals were randomly assigned to six groups, each of which consisted of 10 rats:

Group 1 (normal control group): as the negative control, it was injected with normal saline.

Group 2 (BEP): as the positive control group, received one cycle of 0.5 mg/kg bleomycin, 5 mg/kg etoposide, and 1 mg/kg cisplatin body weight (BW) daily at 10 a.m. by intraperitoneal (IP) injections for 3 weeks. Bleomycin was administered on days 2, 9, and 16, while etoposide and cisplatin were administered on days 1–5 of the 21-day cycle.

Group 3 (NaAL 25): rats were injected with 25 mg/kg of NaAL BW daily at 9 a.m. by IP injections for 42 days. NaAL administration was started 7 days before the start of the BEP injection and continued until 7 days after BEP administration.

Group 4 (NaAL 50): rats were injected with 50 mg/kg of NaAL BW daily at 9 a.m. by IP injections for 42 days. NaAL administration was started 7 days before the start of the BEP injection and continued until 7 days after BEP administration.

Group 5 (BEP + NaAL 25): Rats received the BEP chemotherapy regimen as Group 2 and received 25 mg/kg of NaAL as Group 3.

Group 6 (BEP + NaAL 50): Rats received the BEP chemotherapy regimen as Group 2 and received 50 mg/kg of NaAL as Group 4.

**Figure 1:**
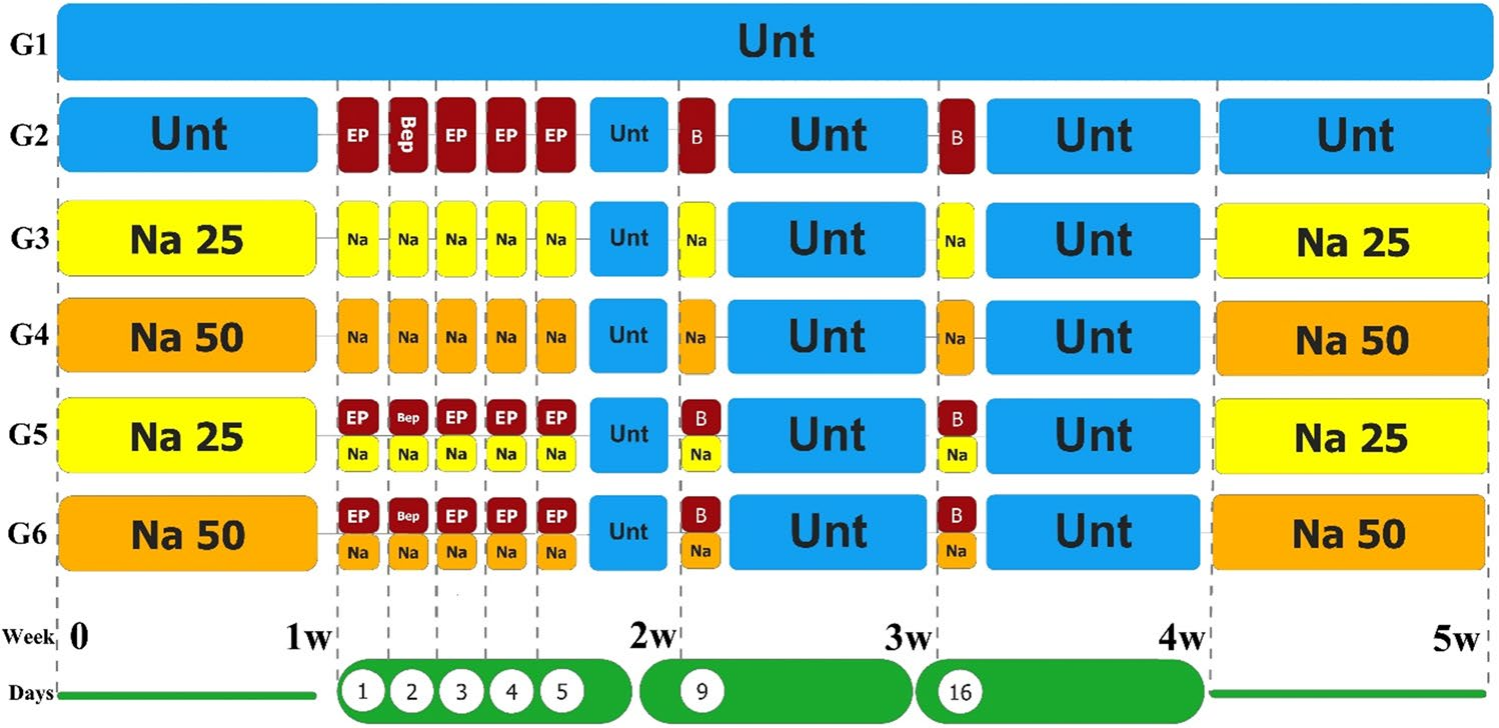
The schematic of experimental treatment plan. BEP chemotherapy regimen was administrated intraperitoneally (IP) for a 21-day period. Sodium alginate was injected (IP) (25 and 50 mg/kg/d) one week before and after BEP administration; and, on days 2, 9 and 16 of BEP therapy, as well. Unt: untreated, E: etoposide, P: cisplatin, B: bleomycin, Na: sodium alginate, W: week.

The doses were chosen based on previous studies and a pilot study^5,37,46,47^. At the end of the study, the animals were weighed and then anesthetized with an intramuscular injection of 50 mg/kg of ketamine and 10 mg/kg of xylazine. Subsequently, 10 mg/100 g of sodium pentobarbital was injected IP for euthanasia. The testes and epididymides were collected by laparotomy for sperm analysis. One testis was fixed for histopathology and immunohistochemical (IHC) evaluation, and the other one was fresh-frozen for oxidant/antioxidant and PCR assessment^5^. All chemicals were purchased from Sigma-Aldrich, USA, unless otherwise specified. The schematic design of the experimental protocols is depicted in Fig. 1.

### Assessment of sperm parameters

Following the treatment period, the animals were weighed and sacrificed. The testes and epididymides were collected by laparotomy for sperm analysis. The epididymis was separated from the testis and the cauda epididymis was cut in 1 mL of pre-warmed Hams F10 medium and incubated in the CO_2_ incubator for 15 min to allow sperm to swim out of the organ. Afterward, sperm count, motility, viability, and morphology were evaluated. Analysis of sperm count was performed via Neobar lam and light microscopy (Olympus CH 30, Tokyo, Japan). Sperm count data were expressed as 10^6^ cells/mL. Furthermore, sperm motility was assessed by counting motile and immotile sperm; and motile sperm were categorized as progressive and non-progressive, as well. Sperm viability was evaluated by Eosin staining for 200 sperm per rat. Furthermore, morphology was assessed by the Papanicolaou test and light microscopy at 1000× magnifications. Sperm with morphological abnormalities in the head and tail were counted and expressed as a percentage of the total sperm count^48,49^.

### Histopathological examination

In the histopathological investigation, the testis was immediately fixed in Bouin's solution. Then, the tissue is embedded in molten paraffin with the aid of metallic blocks, covered with flexible plastic molds, and kept under freezing plates to allow the paraffin to solidify. In the next step, cross-sections (5 µm thickness) of the fixed tissue were cut using a microtome (Leica PM 2125, Germany). Finally, these sections were stained with the hematoxylin and eosin method and visualized under a light microscope (Olympus CH 30, Tokyo, Japan) to examine the possible changes in the organization of germinal epithelium and interstitial connective tissue following the BEP regimen and NaAL treatment^5,48^.

### Stereological estimations

For stereological quantitative estimations, the left testis of each rat was separated and after weighing, fixed in Bouin's solution overnight. The collected testes were cut using the orientator method and a total of 9–10 isotropic uniform random (IUR) slabes were obtained from each testis. The slabes were processed routinely with a tissue processor set (DS2080/H, Iran), and tissue sections with 5 µm thickness were obtained using a rotatory microtome and stained with Hematoxylin and Eosin (H&E)^5,48,50^.

#### Estimating the volume density and total volume

The total volume of testis was considered as a reference volume and determined using the Archimedes method. The fractional volume of the testis sub-compartments was estimated using a point counting probe composed of 40 plus (+) and the relevant formula:

$${\text{V}}_{{\text{v}}} = {{\sum {{\text{P}}_{{{\text{structure}}}} } } \mathord{\left/ {\vphantom {{\sum {{\text{P}}_{{{\text{structure}}}} } } {\sum {{\text{P}}_{{{\text{total}}}} } }}} \right. \kern-0pt} {\sum {{\text{P}}_{{{\text{total}}}} } }}$$

The “ΣP structure” was the sum of the points hitting the desired structures and the “ΣP total” was the sum of the points hitting the whole section. The volume density of the examined structures was multiplied by the reference volume to estimate their absolute volume^5,50^.

#### Estimating the length of the seminiferous tubules

At first, the length density was estimated by the formula introduced by Howard and Reid^5^. Afterward, the total length was estimated by multiplying the length density by the reference volume. Then, the diameter of the tubules was measured perpendicular to the long axis, where the tubule was at the widest point. Finally, an average of 100 profiles/testis were counted^5^.

#### Germinal epithelium height estimation

The germinal epithelial height of seminiferous tubules was calculated quantitatively by dividing the volume density by the surface density of tubules. The diameter of the seminiferous tubules was measured perpendicular to the long axis, where the tubule was at the widest point. Finally, an average of 200 tubules/testis was considered^5^.

#### Estimation of the sperm’s tail length

The epididymis was separated from the testis, and the cauda epididymis was cut in 1 ml of pre-warmed Ham's F10 medium. In the next step, the medium was incubated in a CO_2_ incubator for 15 min to allow sperm to swim out of the organ. Afterward, a drop of this suspension was placed on a microscopic slide, and a smear was prepared by the eosin-nigrosin staining method. Then, the sperm tail length was estimated through a Merz gride, as illustrated by the following formula:

$$L = {{\sum L } \mathord{\left/ {\vphantom {{\sum L } {\sum N }}} \right. \kern-0pt} {\sum N }}$$

**Figure 2:**
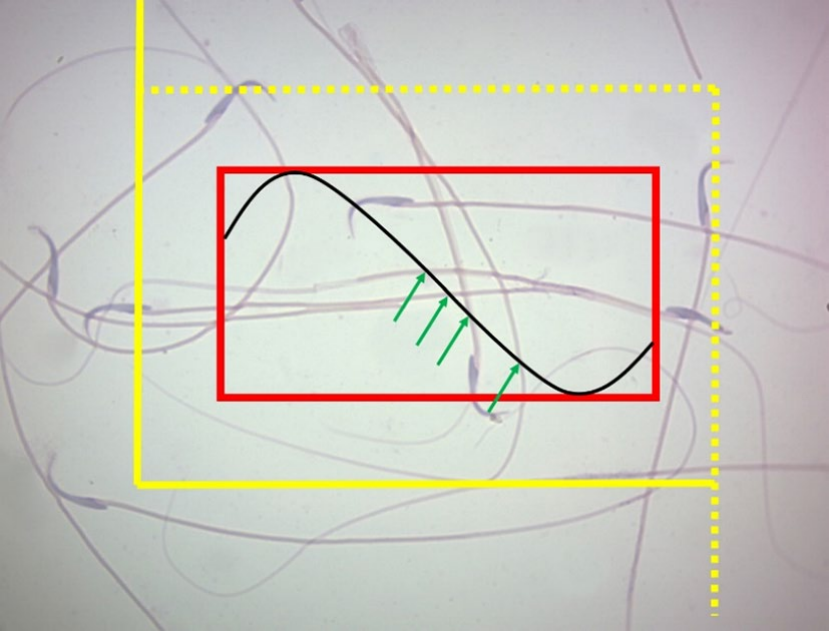
A test system composed of two elements (an unbiased counting frame and a basic tile with a Merz grid inside it was superposed on the image. If the sperm’s head was lay inside the frame and did not touch the forbidden (continued) lines, it was sampled (here 2 sperms). The sperm’s tail length was estimated using the following formula: ΣL = (π/2)·(1/asf)·(a/l)·ΣI, where, ‘‘a/l’’ was the Merz grid constant, ‘‘asf’’ was the area of the basic tile divided by the area of the counting frame, and ‘‘ΣI’’ was the summation of the intersections of the tails with the semicircles (arrowheads; here 4). The total estimated length was divided by the number of counted sperm’s to achieve tail length per sperm.

“ΣL" was the sum of the intersections of the tail of the sperm with the Merz grid and "ΣN" was the total number of the counted sperms in the counting frame. Totally 100–200 sperm heads were counted in each animal (Fig. 2)^5,51,52^.

### Testosterone level in serum

The levels of testosterone were measured by rat testosterone ELISA kits (Moonblind Inc., USA) using the ELISA technique, according to the manufacturer’s instructions. The sensitivity of the testosterone ELISA kits was 0.026 ng/ml^5^.

### Nitro-oxidative stress evaluation

#### Assessment of total antioxidant capacity (TAC) in testicular tissue

Spectrophotometer analysis with the aid of a colorimetric assay kit (Naxifer™, Navand Salamat Co., Iran) was used to evaluate the concentrations of testicular levels of ferric-reducing antioxidant power (FRAP). This procedure is based on the ability of testis lysis to reduce iron III (Fe^3+^) to iron II (Fe^2+^) in the presence of 2,4,6-Tripyridyl-S-triazine (TPTZ). A complex with a blue color and maximum absorbance appeared at 593 nm with a reaction of Fe^2+^ and TPTZ. Finally, the values were expressed as nanomoles of Fe^2+^ equivalents per wet tissue weight (nmol/mg Pro)^5,53^.

#### Assessment of lipid peroxidation (LPO) levels in testicular tissue

To measure the lipid peroxidation products in testis tissue samples, malondialdehyde (MDA) was measured according to the TBA reaction by a Nalondi™ assay kit (Navand Salamat Co., Iran). In brief, 0.2 ml of the aliquots of the homogenate was mixed with 0.8 ml working solution (which contains phosphoric acid and TBA) and then added to the samples. Afterward, the samples were heated at 90 °C for 45 min and cooled on ice. TBARS levels can be detected by absorbance at a wavelength of 532 nm using a standard curve of MDA. Eventually, TBARS were expressed as MDA equivalents per wet tissue weight (gm)^5,54^.

#### Assessment of Nitric oxide (NO) levels in testicular tissue

The total NO content of the homogenized testis was measured according to the Griess reaction, which is a procedure provided by the manufacturer (Natrix™ assay Kit; Navand Salamat Co., Iran). In the Griess reaction, NO rapidly converts into nitrite, which is an acidic environment, and then converts into HNO_2_. After adding Sulfanilamide, HNO_2_ forms a diazonium salt that reacts with *N*-(1-Naphthyl) ethylenediamine dihydrochloride to form an azo dye, which is measured at 570 nm. Finally, The NO content of the examined organs was reported in nmol/mg protein in samples^5,53^.

### Quantitative real-time polymerase chain reaction analysis

Total RNA was extracted with the RNX-Plus Solution Kit (Sinaclone, Iran). The quality and quantity of the extracted RNA were controlled by the UV/Visible Spectrophotometer (Jenway, Malaysia). Regarding the concentration of the extracted RNA and the maximum capacity of the cDNA synthesis kit (Sinaclone, Iran), the extracted RNA was applied to cDNA synthesis immediately. The quantitative real-time PCR was performed with a real-time thermal cycler Rotor-Gene Q (Qiagen, Germany) and the SYBR Green Master Mix (Ampliqon, Denmark). The PCR reactions for mRNA expression consisted of 95 °C for 10 min (denaturing cycle) followed by variable amplification cycles (38–42 cycles) at 90 °C for 15 s (annealing cycle) and 72 °C for 20 s (extension cycle). The sequences of all the genes are listed in Table 1, and *β-actin* was used as a housekeeping gene^5^.

**Table 1:** Sequences of the primer pairs used for RT-PCR.

Gene symbols	Forward sequences	Reverse sequences	Refs.
Bcl-2	5′-CTGGTGGACAACATCGCTCTG-3′	5′-GGTCTGCTGACCTCACTTGTG-3′	^55^
Bax	5′-TTCATCCAGGATCGAGCAGA-3′	5′-GCAAAGTAGAAGGCAACG-3′	^55^
p53	5′-ATGGAGGAGTCACAGTCGGATA-3′	5′-GACTTCTTGTAGATGGCCATGG-3′	^56^
Caspase-3	5′-GGTATTGAGACAGACAGTGG-3′	5′-CATGGGATCTGTTTCTTTGC-3′	^55^
β-actin	5′-AAGTCCCTCACCCTCCCAAAAG-3′	5′-AAGCAATGCTGTCACCTTCCC-3′	^55^

### Immunohistochemical (IHC) assay for *p53* and *Bcl-2* and* TNF-α*

The right testis, after separating the surrounding adipose tissue, was fixed in Bouin's solution for 72 h. The obtained sections were incubated overnight at 65 °C and incubated in tris buffer for antibody retrieval (95 °C for 20 min). In the next step, to block endogenous peroxidase, slides were placed in 5% BSA and incubated in 3% H_2_O_2_. Afterward, Biotinylated rabbit anti-mouse IgG antibody anti-*p53* (dilution, 1:100; Cat#: ab31333) and anti-*Bcl-2* (dilution, 1:500; Cat#: ab196495, Abcam, Cambridge, United Kingdom) were used as the primary antibodies (60 min at room temperature). Then, Biotinylated goat anti-rabbit IgG (Vector Laboratories, Inc.) as the secondary antibody (60 min at room temperature), and streptavidin–horseradish peroxidase (for 30 min) and 1,3-diaminobenzidine (DAB) tetrahydrochloride as evasion solution (for 10 min) were used in the present study. Finally, counterstaining was performed using hematoxylin. All slides were analyzed at 400× magnification using a light microscope (Olympus IX71 microscope, Japan) equipped with a KEcam (KEcam Technologies, Lekki Lagos, Nigeria). The related data were presented as a percent of *p53*, *Bcl2*, and *TNF-α*-positive cells/total cells^5^.

### Statistical analysis

Data analysis was done using SPSS version 26 statistical software. Normality and homogeneity of data were determined by the Kolmogorov–Smirnov test. Quantitative data were reported by mean ± standard deviation and 95% Confidence Interval (CI). Differences between the experimental groups were assessed by one-way ANOVA, followed by Dunnett's post-hoc comparison test. Moreover, the LSD post-hoc comparison test was conducted to compare interventional groups. Differences were considered statistically significant when the *P* value was ≤ 0.05.

### Institutional review board statement

All experimental procedures pursued international guidelines for the care and use of laboratory animals and were approved by the Animal Welfare and Ethics Committee of Razi University and the Kermanshah University of Medical Science, Iran (IR.KUMS.REC.1398.1033).

## Results

### Effects of BEP and sodium alginate on testes and body weight

**Figure 3:**
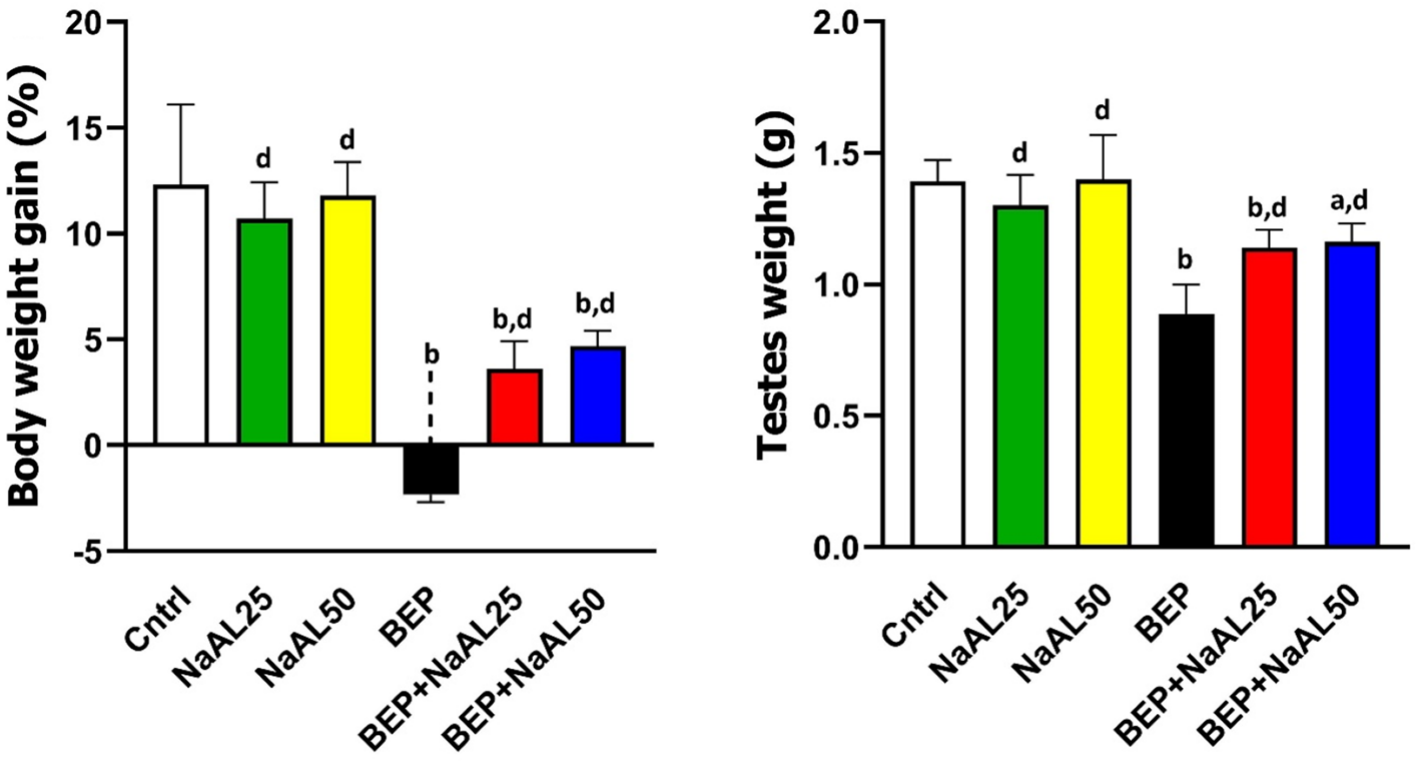
The effect of one cycle of BEP regimen with or without sodium alginate on weight gain (%) and testis weight (g). Cntrl: Control; NaAL 25 and NaAL 50: rats injected with 25 or 50 mg/kg of sodium alginate; BEP + NaAL 25 and BEP + NaAL 50: rats treated with BEP regimen plus 25 or 50 mg/kg of melatonin respectively. ^a^*P* < 0.05 versus Control group; ^b^*P* < 0.01 versus Control group; ^c^*P* < 0.05 versus BEP group; ^d^*P* < 0.01 versus BEP group.

Regarding body weight, there was a non-significant decrease in the animals treated with 25 mg/kg of NaAL compared with the control group (*P* > 0.05). The difference between the BEP-treated group and the other groups was statistically significant (*P* < 0.01). NaAL therapy could significantly improve weight reduction in BEP-injected animals by comparison to the BEP group (*P* < 0.01), yet the differences were still noticeable in comparison to the control group (*P* < 0.01) (Fig. 3). Measurement of testis weight showed that chemotherapy treatment caused a considerable reduction in testis weight relative to the control group (*P* < 0.01). On the other hand, in the BEP-treated groups that received NaAL, the testis weight was significantly higher than the BEP group (*P* < 0.01). Although NaAL maintained the testis weight comparatively, the difference between the BEP + NaAL groups and the control group was still significant (*P* < 0.05) (Fig. 3).

### Effects of BEP and sodium alginate on spermatogenesis

Spermatogenesis was evaluated both histologically and stereologically by estimating the germinal epithelium height of the seminiferous tubules. The results indicated that the germinal epithelium height decreased significantly following BEP administration compared to the control group (*P* < 0.01). This observation indicated that one cycle of the BEP regimen could effectively affect cell division and differentiation in the spermatogenic cell lineage. Nevertheless, there was no significant difference between all groups receiving NaAL with or without the BEP regimen versus the control group (*P* > 0.05), suggesting that NaAL was effective in restoring sperm production (Table 2).

**Table 2:** Total volume (mm^3^) of the testis, seminiferous tubules, interstitial tissue, tubule diameter (µm), the height of the germinal epithelium (µm), total length (m) of seminiferous tubules, and sperm’s tail (µm) in the control and experimental groups.

Parameter	Cntrl	NaAL 25	NaAL 50	BEP	NaAL 25 + BEP	NaAL 50 + BEP
Testis volume (mm^3^)	1081.58 ± 125.54	1124.42 ± 87.85^b,d^	1222.78 ± 61.05^d^	752.76 ± 0.47^b^	767.52 ± 88.82^b^	735.06 ± 106.64^b^
Tubule volume (mm^3^)	896.86 ± 107.44	1292.08 ± 109.78^b,d^	1000.14 ± 58.93^d^	588.46 ± 123.33^b^	541.66 ± 112.47^b^	534.68 ± 95.78^b^
Interstitial tissue volume (mm^3^)	215.62 ± 21.00	230.96 ± 30.08^d^	212.24 ± 3.44^c^	178.38 ± 1.25^a^	199.42 ± 24.13^b^	174.06 ± 14.62
Epithelial height (µm)	123.14 ± 0.99	121.58 ± 3.13^d^	120.74 ± 1.08^d^	87.64 ± 14.27^b^	122.54 ± 5.12^d^	121.54 ± 2.45^d^
TTubule diameter (µm)	325.04 ± 10.49	318.00 ± 11.41^d^	318.28 ± 2.60^d^	250.80 ± 1.24^b^	275.58 ± 32.79^b,c^	279.10 ± 16.23^b,c^
TTubule length (m)	21.34 ± 1.84	20.20 ± 0.70^d^	20.64 ± 1.33^d^	17.58 ± 0.58^b^	18.64 ± 1.42^a^	19.42 ± 1.86^c^
Sperm’s tail length (µm)	30.80 ± 1.24	29.58 ± 2.21^d^	30.10 ± 1.87^d^	24.81 ± 1.18^b^	25.34 ± 1.96^b,d^	29.15 ± 1.64^a,d^

Data were expressed as mean ± SD (n = 5). Cntrl: Control; NaAL 25 and NaAL 50: rats injected with 25 or 50 mg/kg of sodium alginate; BEP + NaAL 25 and BEP + NaAL 50: rats treated with BEP regimen plus 25 or 50 mg/kg of melatonin respectively.

^a^*P* < 0.05 versus control group.

^b^*P* < 0.01 versus control group.

^c^*P* < 0.05 versus BEP group.

^d^*P* < 0.01 versus BEP group.

**Figure 4:**
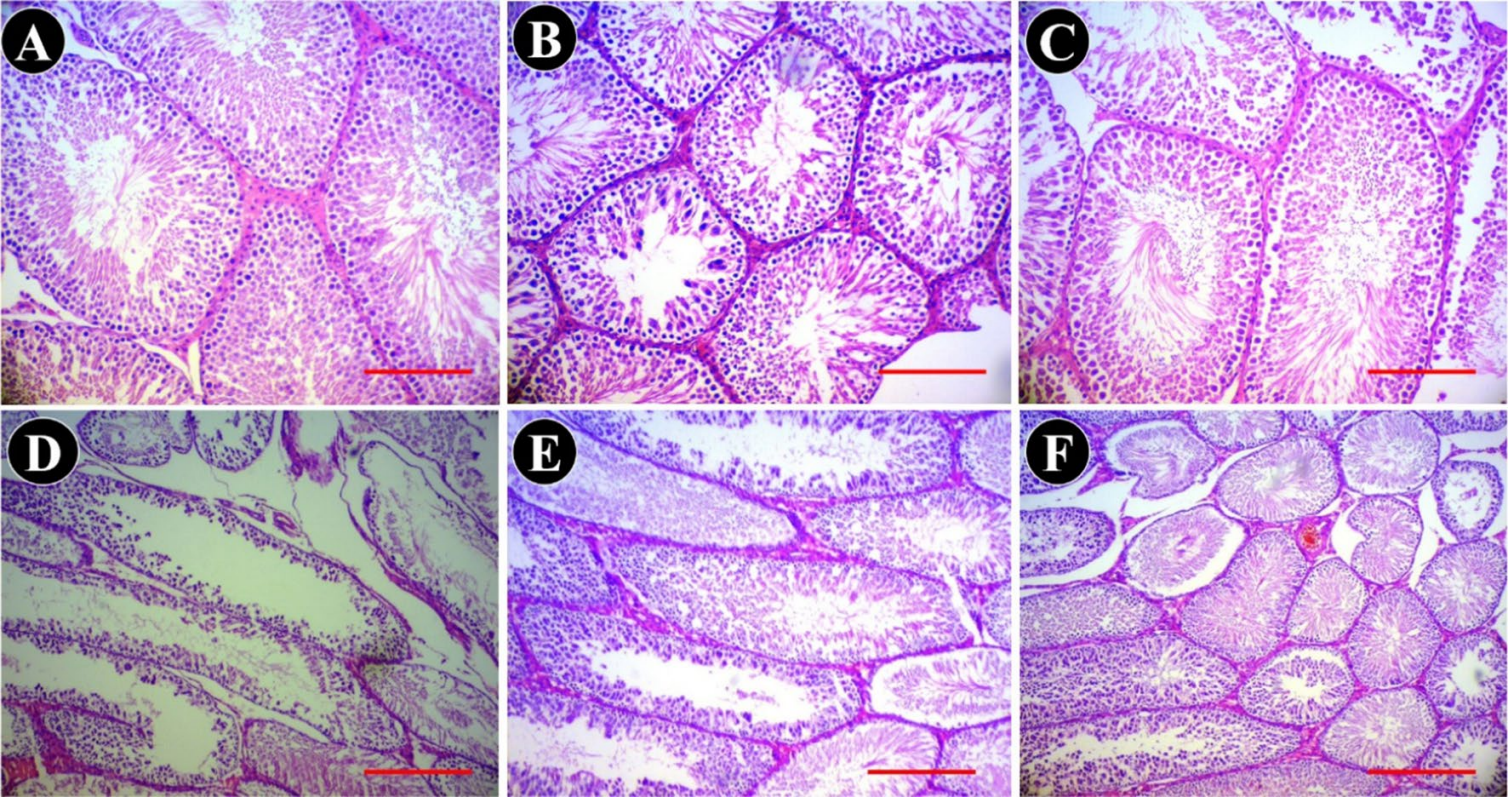
Light microscope micrograph of the testis in the controls and experimental groups. (**A**) normal structure of the germinal epithelium and interstitial tissue are seen in the control group. (**B**) and (**C**) testicular tissue in the control groups treated with 20 and 50 mg/kg of NaAL, respectively, show normal architecture of the testis. (**D**) testicular tissue in the BEP-treated rats showing destructive changes such as atrophic (yellow stars), necrotic (res stars) tubules, and degeneration of interstitial connective tissue (**E**,**F**) testis structure in the BEP-treated rats received 20 and 50 mg/kg of NaAL, respectively, showing improvement of testicular tissue toward normal structure (H&E staining, 100×, scale bar = 100 µm).

From a histopathology point of view, the histological organization of the testis in control groups that received normal saline or NaAL was normal, and no sign of cell apoptosis, necrosis, or tubular atrophy was seen in the seminiferous tubules or interstitial tissue (Fig. 4A–C). Nonetheless, in confirmation of the obtained stereological data, the testis in BEP-treated animals showed a disrupted germinal epithelium in seminiferous tubules associated with congestion in interstitial space. Nectoric and atrophic tubules were scattered throughout the testicular section (Fig. 4D). These pathological changes caused by the BEP regimen were alleviated by NaAL therapy to a considerable degree (Fig. 4E,F).

### Effects of BEP and sodium alginate on testosterone secretion

**Figure 5:**
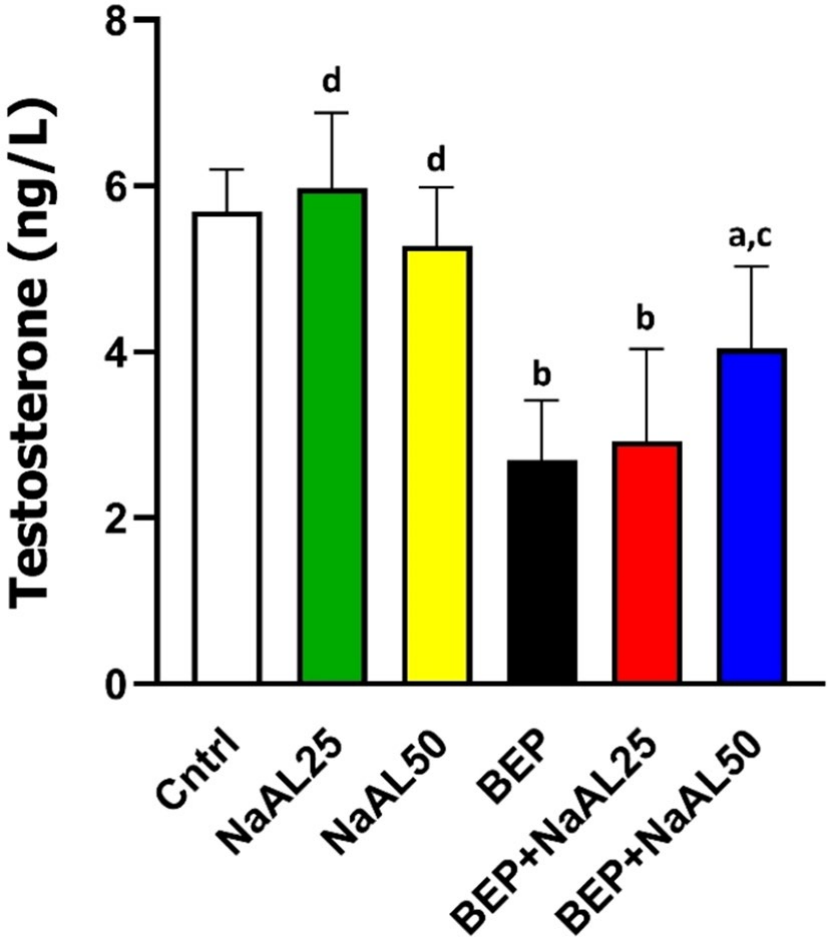
The effect of one cycle of BEP regimen with or without sodium alginate on testosterone level in the controls and experimental groups (n = 5). Cntrl: Control; NaAL 25 and NaAL 50: rats injected with 25 or 50 mg/kg of sodium alginate; BEP + NaAL 25 and BEP + NaAL 50: rats treated with BEP regimen plus 25 or 50 mg/kg of melatonin respectively. ^a^*P* < 0.05 versus Control group; ^b^*P* < 0.01 versus Control group; ^c^*P* < 0.05 versus BEP group; ^d^*P* < 0.01 versus BEP group.

As presented in Fig. 5, the testosterone level was significantly decreased in the BEP-injected animals as compared with the control groups (*P* < 0.01). Even so, in the animals treated with 25 mg/kg of NaAL, the testosterone level was slightly higher than that in the control group (*P* > 0.05). Co-administration of the BEP regimen and 50 mg/kg of NaAL resulted in higher production and secretion of testosterone in the experimental groups in comparison to the BRP group (*P* < 0.05), however, the difference was still significant as compared to the control group. Interestingly, co-administration of 25 mg/kg of NaAL was not capable of enhancing testosterone levels significantly (*P* > 0.05) (Fig. 5).

### Effects of BEP and sodium alginate on sperm parameters

**Figure 6:**
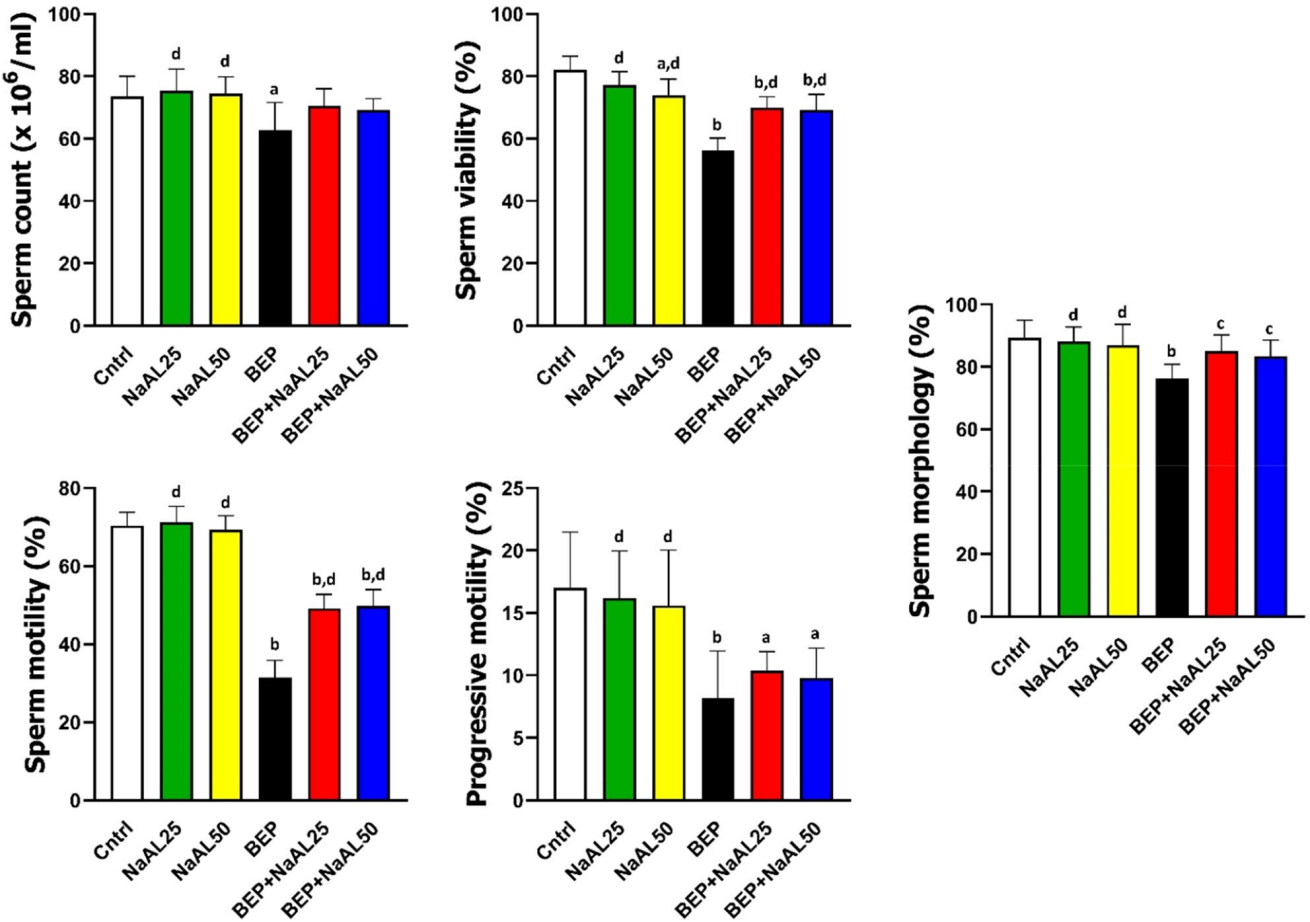
The effect of one cycle of BEP regimen with or without sodium alginate on sperm count, sperm viability, sperm motility, progressive motility, and sperm morphology in controls and experimental groups (n = 5). Cntrl: Control; NaAL 25 and NaAL 50: rats injected with 25 or 50 mg/kg of sodium alginate; BEP + NaAL 25 and BEP + NaAL 50: rats treated with BEP regimen plus 25 or 50 mg/kg of melatonin respectively. ^a^*P* < 0.05 versus Control group; ^b^*P* < 0.01 versus Control group; ^c^*P* < 0.05 versus BEP group; ^d^*P* < 0.01 versus BEP group.

**Figure 7:**
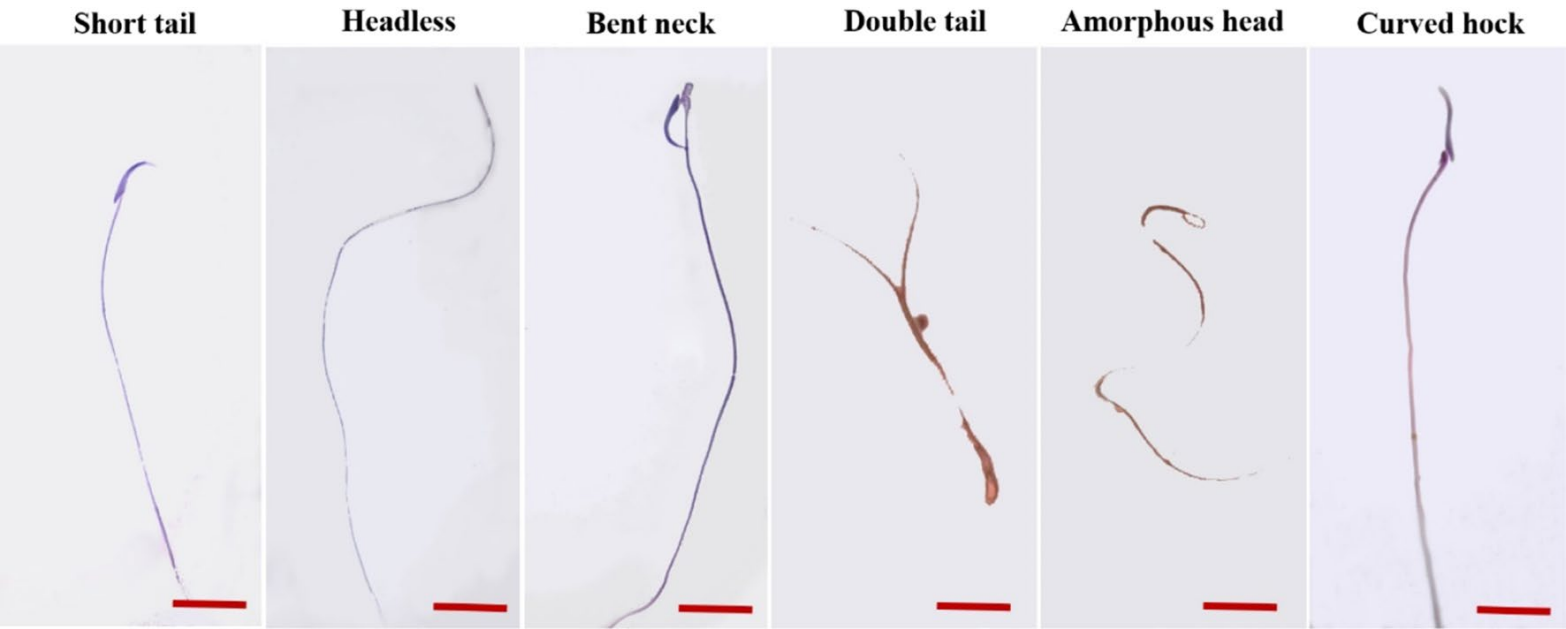
Different abnormalities observed in experimental rats after one cycle of BEP regimen and one week recovery period. Papanicolaou test, scale bar = 5 µm.

As we observed, BEP chemotherapy significantly decreased the sperm count after 21 days compared to the control group (*P* < 0.05), whereas there was no significant difference between the control group and those who received NaAL alone or in combination with BEP (*P* > 0.05). Additionally, sperm motility evaluation showed a significant decrease in sperm motility, especially progressive motility, in BEP-injected rats as compared to the control group (*P* < 0.01). Co-administration of NaAL significantly improved sperm total motility in comparison to the BEP-treated group (*P* < 0.01), while it was still substantially lower than that of the control group (*P* < 0.01). More notably, contrary to what we expected, the administration of NaAL was not able to effectively enhance progressive motility in comparison to the BEP group (*P* > 0.05). Furthermore, the BEP-treated group showed remarkable growth in the number of dead sperm as compared to the control and NaAL-treated ones (*P* < 0.01). In contrast, NaAL administration significantly enhanced sperm survival and sperm viability (*P* < 0.01). Based on sperm morphology investigation, the BEP regimen induced significant abnormal morphology in sperm relative to the control group (*P* < 0.01). However, NaAL therapy could substantially increase the levels of spermatozoa with normal morphology (*P* < 0.05) (Figs. 6 and 7).

### Effects of BEP and sodium alginate on the nitro-oxidative status of the testis

**Figure 8:**
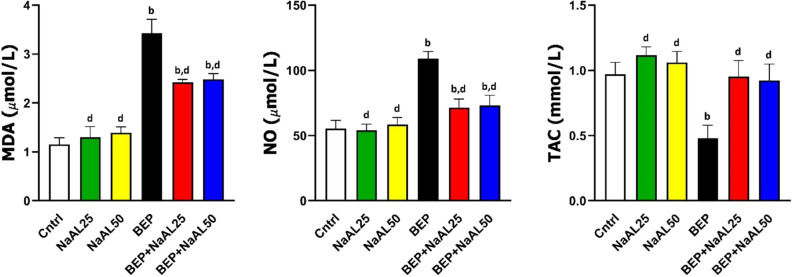
The effect of one cycle of BEP regimen with or without sodium alginate on testicular levels of malondialdehyde (MDA), nitric oxide (NO), and total antioxidant capacity (TAC) in the controls and experimental groups (n = 5). Cntrl: Control; NaAL 25 and NaAL 50: rats injected with 25 or 50 mg/kg of sodium alginate; BEP + NaAL 25 and BEP + NaAL 50: rats treated with BEP regimen plus 25 or 50 mg/kg of melatonin respectively. ^a^*P* < 0.05 versus Control group; ^b^*P* < 0.01 versus Control group; ^c^*P* < 0.05 versus BEP group; ^d^*P* < 0.01 versus BEP group.

The nitro-oxidative status of the testis was investigated by examining MDA, NO, and TAC levels. The MDA and NO levels of the testicular tissue were significantly increased following BEP administration as compared to the control group (*P* < 0.01). The co-treatment of NaAL and BEP regimen considerably diminished MDA and NO levels in the experimental groups; however, there was still a significant difference between the treatment groups and control groups (*P* < 0.01). In addition, the testicular levels of TAC were dramatically reduced in BEP-treated rats in comparison to the control ones (*P* < 0.01). While NaAL could significantly improve the TAC levels compared to the BEP group (*P* < 0.01), there was no significant difference between the control and NaAL-treated groups (*P* > 0.05) (Fig. 8).

### Effects of BEP and sodium alginate on apoptotic-related genes

**Figure 9:**
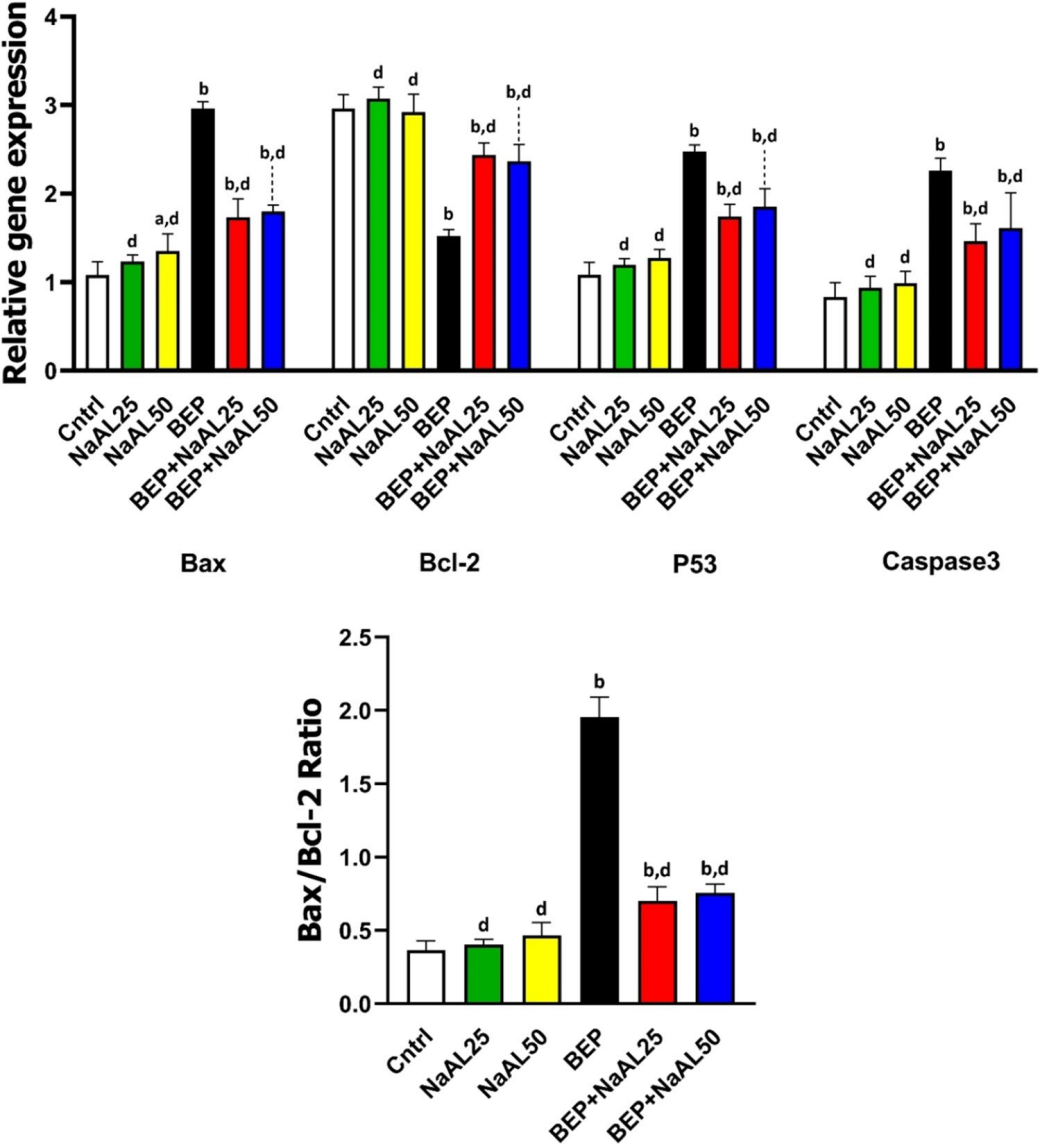
The effect of one cycle of BEP regimen with or without sodium alginate on Bax, Bcl-2, *Caspase-3* (C3), and p53 genes expression and also Bax/Bcl-2 ratio of testis in controls and experimental groups. Cntrl: Control; NaAL 25 and NaAL 50: rats injected with 25 or 50 mg/kg of sodium alginate; BEP + NaAL 25 and BEP + NaAL 50: rats treated with BEP regimen plus 25 or 50 mg/kg of melatonin respectively. ^a^*P* < 0.05 versus Control group; ^b^*P* < 0.01 versus Control group; ^c^*P* < 0.05 versus BEP group; ^d^*P* < 0.01 versus BEP group.

To further elucidate the mechanism of action of BEP and NaAL, we examined apoptotic pathways. PCR results showed that pre-apoptotic genes including *Bax*, *Caspase-3*, and *p53* were significantly up-regulated (*P* < 0.01), and expression of the *Bcl-2* gene was down-regulated in the testicular cells after one cycle of the BEP regimen (*P* < 0.01). Herein, a significant reduction was observed in the expression of the *Bax*, *Caspase-3*, and *p53* genes when 25 and 50 mg/kg of NaAL were associated with the BEP chemotherapy (*P* < 0.01). Additionally, co-administration of NaAL with the BEP regimen resulted in an increase in *Bcl-2* gene expression (*P* < 0.01). Interestingly, the expression of the *Bcl-2* gene in NaAL 25-treated rats was slightly higher than in control rats (*P* > 0.05). Consequently, these alterations led to a marked increase in the *Bax*/*Bcl-2* ratio (*P* < 0.01), a critical phenomenon prior to apoptosis initiation that was meaningfully alleviated by NaAL therapy (*P* < 0.01). Accordingly, NaAL injection suppresses apoptosis levels induced by BEP chemotherapy in testicular cells by down-regulating pro-apoptotic genes and up-regulating the anti-apoptotic gene (Fig. 9).

**Figure 10:**
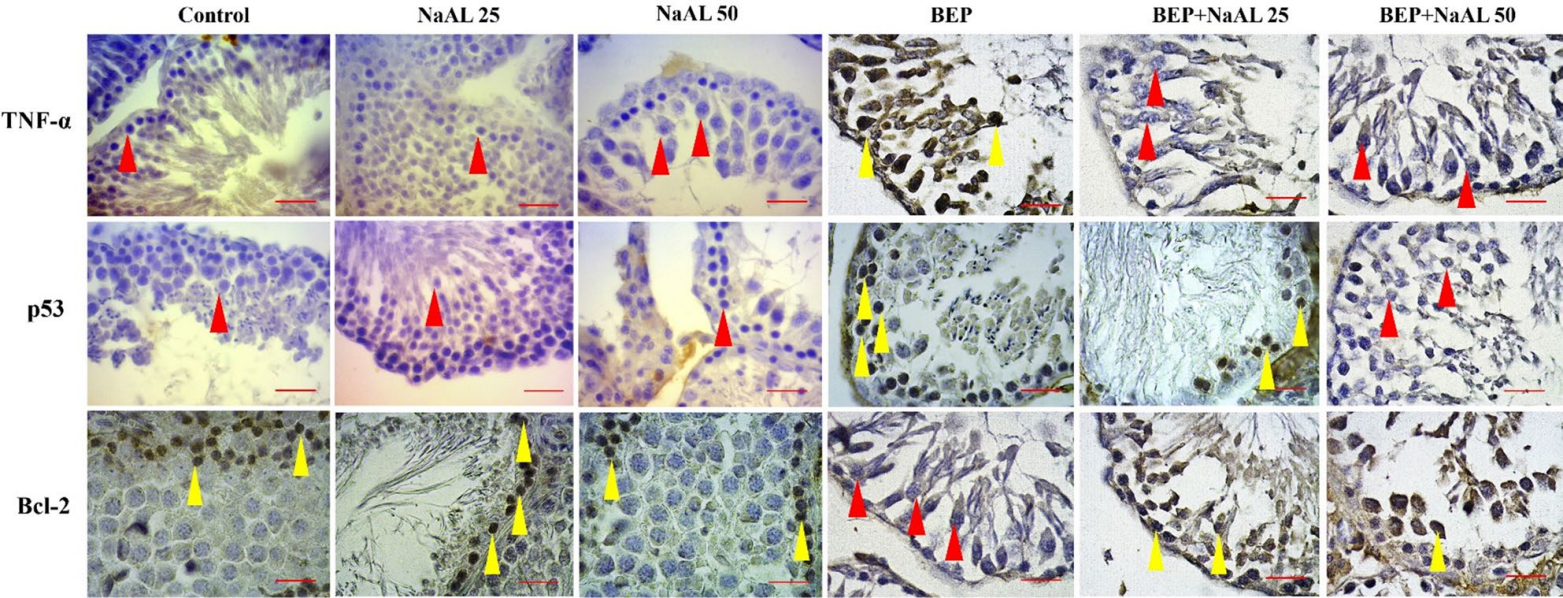
The effect of one cycle of BEP regimen with and without sodium alginate on TNF-α, p53 and Bcl-2 expression in the testis. Intense labeling of p53 and TNF-α are seen in spermatogonia and primary spermatocyte of the BEP-treated group, while the expression in other groups that received NaAL is down-regulated. The expression of Bcl-2 in the control and NaAL-treated groups is remarkably positive, yet in the BEP-treated group, the testicular cell demntrates a negative immunostaining reaction. Yellow arrowheads: positive immunostaining cells, Red arrowheads: negative immunostaining cells (Scale bar = 50 µm, × 400).

Most importantly, immunohistochemical staining also illustrated that *p53* and *TNF-α* proteins were expressed intensively in BEP-treated rats, which resulted in a considerably higher population of positive cells in this group when compared to the control group (*P* < 0.01). Accordingly, *Bcl-2*-positive cells decreased significantly following the BEP therapy regimen compared to the control group (*P* < 0.01). Co-treatment of 25 and 50 mg/kg of NaAL with the BEP regimen significantly modulated the population of *p53* and *TNF-α* positive cells and substantially improved *Bcl-2* protein expression in testicular cells relative to the BEP-treated group (*P* < 0.01) (Figs. 10) (Table 3).

**Table 3:** HSCORE estimation of *Bcl-2*, *p53* and *TNF-α* expression in the control and experimental groups.

Gene	Cntrl	NaAL 25	NaAL 50	BEP	NaAL 25 + BEP	NaAL 50 + BEP
P53	24.15 ± 2.91	24.81 ± 2.52^d^	25.17 ± 3.88^d^	75.45 ± 18.41^b^	37.54 ± 2.41^b,d^	43.77 ± 3.28^b,d^
TNF-α	31.91 ± 8.14	25.18 ± 1.61^d^	31.49 ± 2.39^d^	83.12 ± 6.93^b^	31.85 ± 3.57^d^	35.29 ± 1.53^d^
Bcl-2	88.5 ± 5.2	83.12 ± 4.87^d^	78.44 ± 3.66^d^	31.11 ± 12.9^b^	64.14 ± 4.28^b,d^	71.59 ± 3.72^b,d^

Data were expressed as mean ± SD (*n* = 5). Cntrl: Control; NaAL 25 and NaAL 50: rats injected with 25 or 50 mg/kg of sodium alginate; BEP + NaAL 25 and BEP + NaAL 50: rats treated with BEP regimen plus 25 or 50 mg/kg of melatonin respectively.

^a^*P* < 0.05 versus control group.

^b^*P* < 0.01 versus control group.

^c^*P* < 0.05 versus BEP group.

^d^*P* < 0.01 versus BEP group.

## Discussion

Testicular damage and long-term infertility are two of the main complications of cisplatin-based chemotherapy regimens such as bleomycin, etoposide, and cisplatin (BEP). Previous studies suggest that the gonadotoxicity of anti-cancer drugs can be directly attributed to their adverse effects on testicular antioxidants and apoptosis status^5,24,46,57,58^. In this regard, numerous studies have investigated the beneficial effects of various exogenous antioxidants such as L-ascorbic acid, selenium, zinc, and melatonin on testicular injuries induced by chemotherapeutic drugs^5,46,58,59^. Sodium alginate (NaAL) is a sea salt and water-soluble marine polysaccharide that has been extensively investigated for its antioxidant effects and free radical scavenging capabilities in vitro, yet this antioxidant has been used much less in vivo research^41,43,44^. To the best of our knowledge, for the first time, the present work demonstrates the therapeutic efficacy of NaAL on testes injuries following BEP chemotherapy by enhancing anti-oxidative, anti-inflammatory, and anti-apoptotic defenses in the testis.

Our investigation demonstrated that BEP therapy resulted in significant body weight loss in rats, which is probably due to the systematic toxicity caused by the combination of bleomycin, etoposide, and cisplatin drugs^5,46^. Consistent with a recent study, NaAL treatment significantly improved body weight gain in rats, probably through inhibiting systematic toxicity and increasing rat appetite^5,39^.

The present work showed that the BEP regimen induced a considerable reduction in the testis weight, which was confirmed by stereological estimations. Accordingly, not only the absolute volume of the seminiferous tubules but also the interstitial space, which contains Leydig cells, was significantly diminished in BEP-treated rats. Germinal epithelium height, which is one of the most important stereological parameters representing the functional status of the testis, was also reduced in parallel to the testis volume decrease. Therefore, the reduction of the seminiferous tubule volume following BEP can be attributed to the decrease or interruption in spermatogenesis, leading to a reduction in the germinal epithelium height and the diameter of the testis tubules^46,60^. Due to the loss of Leydig cells observed in histological observation, a remarkable reduction in testosterone level was expected. Considering that the intratesticular concentration of testosterone is necessary for spermatogenesis, the spermatogenic cell line can be affected directly via apoptosis and indirectly via disturbances in testosterone secretion and concentration^46,61^. These results are in agreement with recent research showing that NaAL restored germinal epithelium height and increased serum testosterone levels through free radical scavenging properties^41^.

Here, we observed detrimental effects on sperm count, motility, viability, and morphology following one cycle of BEP therapy. Overproduction of ROS is considered to be the main cause of oxidative stress and to serve as the vital mediator of testicular damage and infertility, which here can justify the BEP-induced disturbance of sperm quality and testosterone levels^5,23,29,62^. Conversely, sperm quality and parameters were restored to normal levels in those rats who received NaAL. Our observations were in agreement with in vitro studies documented that adding alginate to sperm cryopreservation medium effectively enhanced SOD and GSH-Px activities and decreased MDA levels in post-thaw boar spermatozoa. More importantly, NaAL enhanced the motility, acrosomal integrity, and mitochondrial activity of post-thaw sperm^28,43,44,63^. Additionally, Kumar et al.^44^ indicated that NaAL preserves sperm plasma membrane integrity and fluidity, which can justify our results regarding the beneficial effects of NaAL on sperm total and progressive motility.

Consistent with previous studies, our results demonstrated that the BEP administration resulted in a significant elevation in MDA and NO levels and a reduction in TAC levels in the testis^5,57^. Other studies also confirmed the massive production of free radicals and lipid peroxidation following BEP therapy in the testicular tissue and other organs^57,61,64,65^. The main cause of infertility in men is oxidative stress because testicular tissue contains very large amounts of fatty acids, has very high cell division, and has high oxygen consumption^66–68^. The results attained in our study affirmed that co-administration of the NaAL and BEP regimen improved the oxidative status of the testis by increasing TAC levels and modulating MDA and NO levels. NaAL markedly reduced the concentration of MDA, which indicates that alginate effectively prevents lipid peroxidation in the testis, corroborating the results of previous studies^41,44^. Indeed, sodium alginates, not only by containing antioxidant compounds but also by acting as free radical scavengers, protect cells from oxidative stress and improve testis function^43,44,69^.

According to the obtained results, BEP therapy led to a meaningful rise in *TNF-α* expression, indicating the initiation of an inflammatory process in the testis. Yet, NaAL administration in the experimental groups exhibited an anti-inflammatory effect by alleviating *TNF-α* protein expression towards normal levels. This observation provides evidence that alginate could protect the testis through not only antioxidant effects but probably anti-inflammatory pathways. This finding aligns with previous studies demonstrating the anti-inflammatory properties of alginate slats. For instance, Mirshafiey et al.^37^ reported the role of NaAL in treating immune complex glomerulonephritis in a rat model. Alginate is a polyuronic acid molecule and has anti-inflammatory properties. Accordingly, NaAL could inhibit hyaluronidase activity and mast cell degranulation, thereby protecting tissue and cells from mast cells containing histamine. Another study revealed that alginate in combination with allicin and ascorbic acid modulated *TNF-α*-induced ICAM (intercellular adhesion molecule-1) and suggested that this compound could be useful in the treatment of various inflammation-related disorders associated with an increase in endothelial leukocyte adhesion molecules^70^.

Consistent with our previous studies, these results demonstrated that BEP therapy increased the apoptosis rate in the spermatogenic line cells of the testis^5^. The promotion in the expression of *Bax*, *Caspase-3*, and *P53* genes was associated with down-regulation of the *Bcl-2* gene and protein expression. This apoptotic activation can be attributed to a considerable elevation in ROS and reactive nitrogen species (RNS) levels, which agitate the mitochondrial membrane potential and permeability^5,41^. On the other hand, these disturbances in pro-apoptotic and anti-apoptotic gene expression were effectively modulated by the administration of NaAL. This is the first report showing that NaAL inhibits apoptosis in testicular cells, which brings about an improvement in testicular function and spermatogenesis. Earlier reports indicated that alginates oligosaccharides attenuate nitro-oxidative stress and endoplasmic reticulum (ER) stress-mediated apoptosis in neurodegenerative diseases and acute doxorubicin cardiotoxicity^41,42^. Likewise, a more recent study also confirmed that alginate alleviates myocardial reperfusion injury through a similar pathway^40^.

## Conclusion

The present study provides evidence that sodium alginate effectively relieves BEP chemotherapy regimen-induced reproductive toxicity by reducing nitrosative and oxidative stress and regulating the inflammatory and apoptotic processes. More detailed studies would be needed to elucidate the exact mechanisms governing the alleviating effects and pharmacokinetics of sodium alginate. Our results also suggest that sodium alginate may serve clinically as a nutritional supplement to improve fertility impairment in young patients receiving BEP and improve their quality of life.

## Data Availability

The data presented in this study are available on request from the corresponding author. The data are not publicly available due to privacy or ethical restrictions.
